# Updated registry of congenital adrenal hyperplasia at the north pediatric referral centre of Vietnam

**DOI:** 10.1186/1687-9856-2015-S1-P49

**Published:** 2015-04-28

**Authors:** Vu Chi Dung, Bui Phuong Thao, Can Thi Bich Ngoc, Nguyen Ngoc Khanh, Nguyen Phu Dat, Nguyen Thi Hoan, Do Thanh Mai, Maria Craig

**Affiliations:** 1 National Hospital of Pediatrics, Hanoi, Vietnam; 2 School of Women's and Children's Health, UNSW Medicine, Sydney, NSW, Australia

## 

The National Hospital of Pediatrics (NHP), Hanoi, Vietnam is an 1200 bed tertiary referral centre servicing approximately 30 million people from northern provinces of Vietnam. This audit was undertaken to analyze anecdotal reports of increasing patient numbers. Retrospective review of all CAH patients registered at NHP from 1999- 5.2014. Ethical clearance was granted by the NHP Directorate.

At the start of 1999 there were 90 children with CAH managed at NHP. By May 2014 this increased to 715 including 375 (52%) male patients and 340 (48%) female patients. Number of cases with 21α-hydroxylase deficiency (21-OHD), 11β-hydroxylase deficiency and 3β-hydroxysteroid dehydrogenase deficiency was 703 (98.3%); 9 (1.3%) and 3 (0.4%), respectively. Among cases with 21-OHD, 72% were salt wasting and 28% were simple virilisation). Total number of cases representing a more than seven fold increase over 14 years. Number of new cases doubled from 30 to 67 in 2013. Most children (85%) were diagnosed at less than 12 months of age (55% at less than 1 month of age); 70% of all children were younger than 10 years. Formal mortality figures were low (7 known deaths).

The caseload of CAH at NHP has increased since 1999 and additional capacity is needed for patient care. Introduction of NBS would enable more accurate estimation of CAH incidence, reduce infant mortality and minimize trauma to affected infants and their families.

